# Predictors of permanent pacemaker implantation in aortic valve diseases after TAVI with vitaFlow liberty system

**DOI:** 10.3389/fcvm.2023.1277528

**Published:** 2023-09-29

**Authors:** Changlin Ju, Xiangrong Xie, Shengxin Tang, Shiping Cao

**Affiliations:** ^1^ Department of Cardiology, Nanfang Hospital, Southern Medical University, Guangzhou, China; ^2^ Department of Cardiology, The First Affiliated Hospital of Wannan Medical College, Wuhu, China

**Keywords:** transcatheter aortic valve implantation, permanent pacemaker implantation, cardiac conduction block, aortic regurgitation, aortic stenosis

## Abstract

**Introduction:**

Permanent pacemaker implantation (PPI) is a known complication in patients with aortic stenosis following transcatheter aortic valve implantation (TAVI). However, there is limited research on TAVI for pure aortic regurgitation (PAR), and more investigation is needed to determine the occurrence of postoperative cardiac conduction block and the need for PPI in this population. Therefore, this retrospective analysis aimed to evaluate the incidence of cardiac conduction block and the necessity of PPI after TAVI in patients with different types of aortic valve disease, including pure aortic stenosis (PAS), aortic stenosis with regurgitation (ASR), and PAR.

**Methods:**

Clinical data of 100 patients who TAVI were analyzed retrospectively. The incidence of conduction block was assessed, and clinical factors were examined to predict the necessity of PPI.

**Results:**

Cardiac conduction block was found to be a common complication following TAVI, particularly in patients with PAR. PAR was identified as an independent risk factor for requiring PPI. Additionally, first-degree atrioventricular block emerged as a sensitive predictor for PPI in patients with PAR.

**Discussion:**

These findings provide valuable insights into the safety and effectiveness of TAVI, which can help enhance patient management and reduce complications.

## Introduction

After more than two decades of research and development, transcatheter aortic valve implantation (TAVI) has become increasingly common. Previously, TAVI was primarily reserved for individuals with severe aortic stenosis who were deemed high-risk or unsuitable for surgery. However, it is now increasingly employed to treat patients with lower and moderate risk profiles (1), Although TAVI has been used in high-risk patients with pure aortic regurgitation (PAR) in some case series (2), this off-label use still necessitates additional research to ascertain the procedure's safety and the risk of postoperative complications.

To explore these issues, we conducted a retrospective analysis of 100 patients who underwent TAVI at our hospital over the past two years. Specifically, we observed cardiac conduction block in patients with different types of aortic valve disease and explored clinical factors that may predict the need for new permanent pacemaker implantation (PPI) after TAVI. Through this analysis, our aim is to acquire a more comprehensive understanding of the safety and effectiveness of TAVI in diverse patient groups and to pinpoint strategies for reducing potential complications.

## Materials and methods

### Research subjects

This single-center retrospective study involved the analysis of 100 consecutively enrolled patients who received TAVI in our hospital from February 2021 to February 2023. The study samples included 49 males and 51 females, with a mean age of 73.75 ± 7.75 years, ranging from 53 to 87 years old. One week after TAVI, patients were classified into two groups: the PM group (patients with new PPI) and the NPM group (patients without new PPI). According to the type of aortic disease, three subgroups were identified: 33 cases of pure aortic stenosis (PAS), 23 cases of aortic stenosis with regurgitation (ASR), and 44 cases of pure aortic regurgitation (PAR). The inclusion criteria for this study were patients suffering from symptomatic aortic valve disease who had been diagnosed with severe AS, with or without PAR, by echocardiography before surgery or only severe PAR and Society of Thoracic Surgeons (STS) risk score ≥4% (high risk in surgery). Meanwhile, patients with left ventricular thrombus, left ventricular outflow tract obstruction, and anatomical morphology were not suitable for TAVI, contraindications to anticoagulation, and a life expectancy of less than 12 months after correcting valve diseases were excluded from the study. The Ethics Committee of our hospital reviewed and approved this study, and all patients signed informed consent forms before participating in this study.

### TAVI procedure

The TAVI procedure was performed using the VitaFlow Liberty system via the femoral artery. Close monitoring of EKG changes was conducted post-procedure to identify any significant bradycardia necessitating PPI. Skilled interventional cardiologists performed all procedures according to standard care protocols. The size of the prosthesis was selected based on CT scanning measurements of the aortic ring area. The positioning of the aortic valve was guided by angiography and transesophageal echocardiography (TEE), and the valve was released at the level of the right coronary sinus under very fast pacing (≥160 beats/min). Following the procedure, patients received management in accordance with local standard care practices, with subsequent transthoracic echocardiography (TTE) and EKG evaluations conducted at discharge (3, 4).

### Procedural assessment

**Figure 1:**
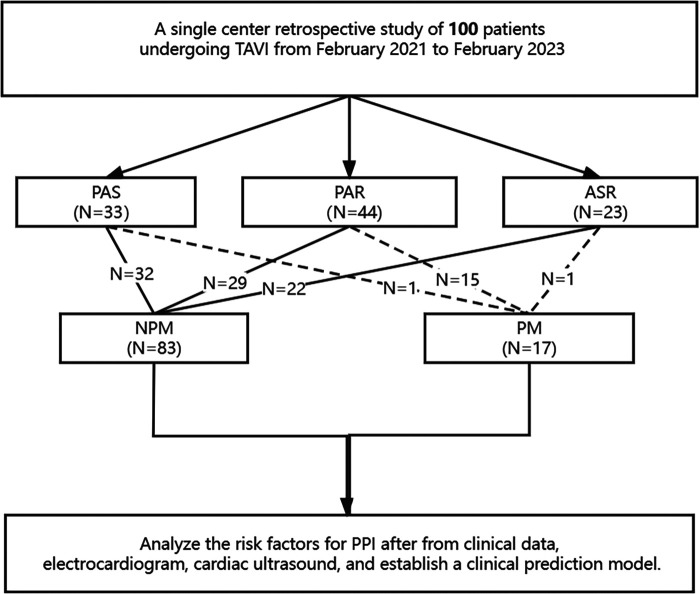
A schematic diagram showing permanent pacemaker implantation after TAVI. PAR, pure aortic regurgitation; PAS, pure aortic stenosis; ASR, aortic stenosis & regurgitation.

All patients underwent TTE and EKG within one week before the interventional procedure. The diameter and wall thickness of each chamber, the diameter of the ascending aorta, and the left ventricular ejection fraction (LVEF) were measured using biplane Simpson. The width of the vena contracta (VC) and the effective regurgitant orifice area (EROA) were measured using PISA. All patients underwent the implantation of a temporary pacemaker via the right internal jugular vein before TAVI. The temporary pacemaker provided rapid ventricular pacing during valve deployment to reduce cardiac output, ensuring the position of the balloon and valve. Continuous monitoring was performed postoperatively to prevent the occurrence of severe cardiac conduction block. If there were no new cardiac conduction blocks within three days after the procedure, the temporary pacemaker was removed. For patients who developed high-degree or complete atrioventricular block (AVB) during or after the procedure and did not recover within one-week, permanent pacemaker was implanted. Additionally, 12-lead EKG exams were performed on all patients before and after the procedure. AV interval, QRS width, and the presence of right bundle branch block (RBBB) and left bundle branch block (LBBB) were recorded before and after the procedure. Patients with type 2 second-degree or advanced AVB were implanted with permanent pacemakers if they did not recover after one week (5) (Figure 1).

### Statistical methods

Statistical software SPSS 26.0 was used for statistical analysis of data. The measurement data were assessed for normality and homogeneity of variance and were expressed as $\bar{\chi }\pm s$, and inter-group comparisons were conducted using the t-test. Categorical variables are presented as counts and percentages and were compared using the *χ*^2^ test or Fisher's exact test, depending on the minimum number of observations. All statistical tests were two-sided, and a *p*-value <0.05 was considered statistically significant.

## Results

### Comparison of clinical parameters

Among the 100 patients who underwent TAVI, 17 patients received PPI after the procedure. There was no statistical difference between the two groups in terms of gender, age, renal function, blood lipid analysis, and the presence of complications such as hypertension, coronary heart disease, type 2 diabetes, atrial fibrillation, obstructive emphysema, mitral insufficiency, and New York heart function classification. However, the valve diameter implanted in PM group was significantly larger compared to the NPM group. Further subgroup analysis revealed a significant difference in pacemaker rate among the AS, ASR, and PAR groups. Specifically, the PAR group had a significantly higher pacemaker rate compared to the other two groups (Table 1).

**Table 1:** Baseline characteristics according to pacing outcome.

Baseline	No-PM (*n* = 83)	PM (*n* = 17)	*p*-value
Age (Y)	72.92 ± 7.87	72.12 ± 8.15	0.706
Gender (male, %)	41 (49.40)	8 (47.06)	0.860
Creatinine (mmol/L)	96.15 ± 68.10	138.19 ± 224.49	0.162
Lipoprotein(a) (mmol/L)	301.51 ± 341.86	461.25 ± 593.39	0.203
Uric acid (mmol/L)	392.41 ± 141.79	415.49 ± 168.06	0.593
Glucose (mmol/L)	5.16 ± 1.13	5.07 ± 0.79	0.766
Cholesterol (mmol/L)	3.82 ± 1.24	4.21 ± 1.26	0.281
Triglyceride (mmol/L)	1.14 ± 0.81	1.33 ± 0.92	0.411
HDL-c (mmol/L)	1.34 ± 0.38	1.46 ± 0.35	0.274
LDL-c (mmol/L)	2.11 ± 0.93	2.34 ± 0.84	0.398
Apo A1 (mmol/L)	1.20 ± 0.40	1.30 ± 0.25	0.400
Apo B (mmol/L)	0.61 ± 0.25	0.66 ± 0.22	0.505
Hypertension (%)	37 (44.05)	11 (64.71)	0.130
CAD (%)	16 (19.28)	4 (23.53)	0.690
Diabetes mellitus (%)	8 (10.84)	2 (11.76)	0.790
Atrial fibrillation (%)	19 (22.89)	4 (23.53)	0.955
Cerebrovascular disease (%)	4 (4.82)	0 (0)	0.356
MR (%)	17 (20.48)	4 (23.53)	0.703
COPD (%)	6 (7.23)	1 (5.88)	0.690
Cancer (%)	3 (3.61)	2 (11.76)	0.160
CRBBB (%)	6 (7.23)	3 (17.65)	0.171
CLBBB (%)	6 (7.23)	8 (47.06)	0.530
NYHA (%)	3.22 ± 0.73	2.94 ± 0.75	0.162
Aortic disease			
PAS	32 (38.55)	1 (5.88)	0.000**
PAR	29 (34.94)	15 (88.24)	
ASR	22 (26.51)	1 (5.88)	
Valve-in-valve (%)	1 (1.20)	6 (35.29)	0.253
Prosthesis size (%)	26.86 ± 2.79	28.41 ± 2.15	0.033*

HDL-c, high-density lipoprotein cholesterol; LDL-c, low-density lipoprotein cholesterol; CAD, coronary artery disease; MR, mitral regurgitation; COPD, chronic obstructive pulmonary disease; CRBBB: complete right bundle branch block; CLBBB: complete left bundle branch block; NYHA, New York Heart Association; AS, aortic stenosis; PAR, pure aortic regurgitation; ASR, aortic stenosis and regurgitation.

* *p* < 0.05, **p < 0.01.

### Comparison of preoperative cardiac ultrasound parameters

Cardiac ultrasound revealed that there were significant differences (*p* < 0.05) in LVPW, sinus of valsalva, IVS, mean pressure gradient, VC and EROA between the PM and NPM groups. However, there were no significant differences in LVEDD, LVEF, AAO, and aortic annulus (*p* > 0.05) between the groups (Table 2).

**Table 2:** UCG characteristics according to pacing outcome.

Parameters	NPM (*n *= 83)	PM (*n *= 17)	*p*-value
LVEDD (mm)	54.51 ± 8.24	57.53 ± 10.56	0.193
LVPW (mm)	11.43 ± 2.25	10.12 ± 1.54	0.024*
Aortic annulus (Φ, mm)	21.22 ± 7.34	24.43 ± 3.17	0.081
Sinus of Valvsalva (Φ, mm)	31.28 ± 93	37.47 ± 6.36	0.041*
AAO (Φ, mm)	40.57 ± 9.54	39.94 ± 5.08	0.794
IVS (mm)	12.37 ± 3.02	10.59 ± 2.18	0.023*
LVEF (%)	54.16 ± 10.77	54.65 ± 8.19	0.860
Mean PG (mmHg)	29.76 ± 26.85	4.35 ± 11.82	0.000**
VC (mm)	4.00 ± 3.97	7.59 ± 2.93	0.001**
EROA (cm^2^)	5.15 ± 5.10	9.05 ± 2.90	0.003**

LVEDD, left ventricular end-diastolic dimension; LVPW, left ventricular posterior wall; AAO, Aorta ascendens, IVS, left ventricular posterior wall; LVEF, left ventricular ejection fraction; PG, pressure gradient; VC, vena contracta; EROA, the effective regurgitant orifice area.

* *p* < 0.05, **p < 0.01.

### The impact of TAVI on the conduction system

After excluding 17 patients who received new PPI and 23 patients with atrial fibrillation, a total of 60 patients were further analysed. Following TAVI for various types of aortic valve diseases, there were no significant differences in the pre- and post-AV (atrioventricular) interval, QRS width changes, ΔAV interval (post-AV—pre-AV), and ΔQRS (post-QRS—pre-QRS) among the groups (Table 3).

**Table 3:** Pre- and post-TAVI ECG characteristics according to pacing outcome.

Parameters	PAS (*n *= 22)	PAR (*n *= 23)	ASR (*n *= 15)	*p*-value
Pre AV	170.55 ± 32.79	167.00 ± 16.43	175.82 ± 37.07	0.737
Pre QRS	102.45 ± 24.90	101.62 ± 20.94	104.06 ± 17.18	0.950
Post AV	176.91 ± 29.30	179.31 ± 31.97	182.59 ± 37.56	0.867
Post QRS	113.86 ± 29.82	127.00 ± 31.69	115.29 ± 24.40	0.397
ΔAV	6.36 ± 33.54	12.31 ± 30.38	6.76 ± 47.06	0.893
ΔQRS	11.41 ± 35.21	25.38 ± 33.51	11.24 ± 25.11	0.393

ΔAV = Post AV- Pre AV, Δ QRS = Post QRS- Pre QRS.

* *p* < 0.05, **p < 0.01.

### PPI in cardiac conduction block of TAVI

Before TAVI, there were a total of 26 cases with cardiac conduction block, including 9 cases of RBBB, 8 cases of LBBB, and 9 cases of I° AVB. Among these, in the PAR group alone, 3 cases of RBBB, 2 cases of LBBB, and 3 cases of I° AVB underwent PPI after TAVI. However, there were no patients with cardiac conduction block in the PAS and ASR groups who underwent PPI.

After TAVI, there was no significant difference observed between different aortic valve diseases in terms of the occurrence of 16 cases of LBBB and 16 cases of I° AVB. However, four new cases of RBBB were reported, and all of them were in the PAR group, with one case receiving PPI. Additionally, 19 new cases of type II or above AVB were reported, among which 17 cases underwent PPI.

The overall incidence of PPI after TAVI was 17%, but there was a significant difference (*p* < 0.01) among the PAS, PAR, and ASR groups. The incidence of PPI in the PAR group alone was 34.09% (Table 4).

**Table 4:** Conduction block of different aortic valve diseases before and after TAVI.

Parameters	Pre-TAVI			Post-TAVI			
	CRBBB (PM)	CLBBB (PM)	I°AVB (PM)	CRBBB (PM)	CLBBB (PM)	I°AVB (PM)	≥II°IIAVB (PM)
PAS (*n* = 33)	3 (0)	1 (0)	4 (0)	0 (0)	3 (0)	7 (0)	1 (1)
PAR (*n* = 44)	5 (3)	6 (2)	3 (3)	4 (1)	6 (2)	4 (1)	17 (15)
ASR (*n* = 23)	1 (0)	1 (0)	2 (0)	0 (0)	7 (0)	5 (0)	1 (1)
Sum (*n* = 100)	9 (3)	8 (2)	9 (3)	4 (1)	16 (2)	16 (1)	19 (17)
*p*-value	0.691	0.254	0.901	0.071	0.085	0.263	<0.01

### Independent predictors of pacing outcome

As shown in Table 5, univariate and multivariate regression analyses were performed to analyze the relevant indicators with differences between the PM and NPM groups. The following indicators were examined: type of aortic valve disease, sinus of valsalva, EROA, cardiac conduction block, and implanted prosthesis size.

**Table 5:** Independent predictors of the pacing outcome.

Parameters	Univariate			Multivariate		
	*p*-value	OR	95% CI	*p*-value	OR	95% CI
Aortic disease^a^	0.002	5.435	1.825∼16.182	0.019	5.35	1.321∼21.658
LVEDD	0.194	1.041	0.980∼1.105	0.746	1.016	0.924∼1.116
Sinus of Valvsalva	0.047	1.099	1.001∼1.206	0.123	1.082	0.979∼1.196
EROA	0.006	1.181	1.048∼1.332	0.978	0.997	0.809∼1.229
Conduction block	0.287	2.014	0.556∼7.300	0.597	1.486	0.342∼6.454
Prosthesis size	0.039	1.28	1.012∼1.619	0.997	1.001	0.710∼1.410
NYHA Grade	0.164	0.604	0.296∼1.229	0.286	0.628	0.267∼1.476
LVEF	0.858	1.005	0.954∼1.058	0.332	1.039	0.961∼1.123

^a^ Aortic valve disease, 1. Aortic valve stenosis, 2. Aortic valve insufficiency, 3. Aortic valve stenosis with insufficiency; LVEDD, left ventricular end-diastolic dimension; EROA, the effective regurgitant orifice area; LVEF, left ventricular ejection fraction.

Among these indicators, the presence of PAR was found to be an independent risk factor for PPI after TAVI. The odds ratio (OR) was calculated to be 5.350, with a 95% confidence interval (CI) ranging from 1.321 to 21.658. This suggests that patients with PAR are more likely to require pacemaker implantation after TAVI compared to those without AR.

### Sensitivity and specificity analysis of cardiac conduction block

After TAVI, I° AVB has been found to have a sensitivity of 100% and a specificity of 70.7% in predicting the need for PPI in patients with PAR. Additionally, when compared to RBBB and LBBB, I° AVB demonstrates superior predictive ability for PPI in PAR patients (Table 6).

**Table 6:** Sensitivity and specificity analysis of conduction block prediction in patients with aortic regurgitation for new PPI after TAVI.

Variable	True positive (cases)	False positive (cases)	True negative (cases)	False negative (cases)	Sensitivity (%)	Specificity (%)	Accuracy (%)
I°AVB	3	0	29	12	100	70.7	72.7
RBBB	3	2	27	12	60	69.23	76.92
LBBB	2	4	25	13	33.33	65.79	71.05

I°AVB, I degree atrioventricular block; RBBB, complete right bundle branch block; LBBB, complete left bundle branch block.

## Discussion

This study found that cardiac conduction block is commonly observed after TAVI. Moreover, the type of aortic valve diseases, mean pressure gradient, EROA and implanted prosthesis size are related to PPI. The incidence of PPI in PAR patients is 34%, which is an independent factor for PPI after TAVI. Conduction block is indeed a common complication not only in patients with PAS but also in those with PAR who undergo TAVI. Furthermore, the incidence of AVB tends to be higher in these cases. The most common type of cardiac conduction block is newly diagnosed LBBB. Previous research has shown that the incidence rates of LBBB with Edwards SAPIEN and CoreValve ReValving during TAVI are 14.8% and 25%, respectively (6). The meta-analysis has also demonstrated that newly diagnosed LBBB ranges from 13.3% to 37% after TAVI (6). This study found that the incidence of LBBB after TAVI with VitaFlow Liberty^™^ was lower at 16% compared to 77% with Lotus^™^ Valve System (7) and 52% with Edwards SAPIEN XT 100 (8). There were no significant differences observed in cardiac conduction block among different aortic valve diseases after TAVI. Previous studies have indicated that nearly 90% of new LBBB cases occur either during or within 24 h after TAVR, potentially due to mechanical damage to the conduction system during balloon dilation and valve implantation. This injury is often temporary, and some newly diagnosed LBBB cases could recover within hours or days (9, 10). RBBB occurs less frequently than LBBB and may be associated with the PAR group. AVB is another common type of cardiac conduction block following TAVI (11). Approximately 22% of patients who undergo TAVI develop new-onset AVB, which is associated with a five-fold higher risk of permanent AVB requiring a PPI (12). This study found that the incidence of advanced II°AVB was 38.4% after TAVI with VitaFlow Liberty^™^, with a PPI rate of 34.09% in the PAR group. These rates were significantly higher than those observed in the PAS group, which had an AVB incidence of 3.03%, and the PPI rates ranged from 6.1% to 27.3% in PAR patients (2, 13). As for the type of PPI, current reports mainly focus on conventional single- or dual-chamber lead pacemakers. However, patients undergoing TAVI often have poor physical conditions, such as advanced age, frailty, and oral antiplatelet or anticoagulant medications. These factors increase the risk of bleeding and infection associated with traditional pacemakers. In this context, leadless pacemakers may be a preferable choice (14, 15).

Pre-existing cardiac conduction block can be used to predict the risk of PPI after TAVI. Pre-existing RBBB (7, 16–18) and prolonged PR interval (19, 20) were found to be significant predictors for PPI in PAS patients. In this study, 60% of pre-existing RBBB and all pre-existing AV ≥ 200 ms in patients with AR necessitating PPI. Consistent with previous findings, pre-existing LBBB did not predict the need for a pacemaker. The distribution of the His bundle is such that 50% is located on the right side of the membranous septum, 30% on the left side, and 20% within the septum. TAVI can potentially cause injury to the His bundle in the latter two cases, resulting in complete AVB (8, 21). This can reasonably explain that LBBB is not a risk factor for predicting PPI but rather the most common arrhythmia after TAVI. Our findings demonstrate that PR prolongation is the most sensitive predictor for PPI after TAVI.

This study also shows that the anatomy of the left ventricular outflow tract (LVOT) and the size of the valve diameter are related to PPI. Similar to previous studies, patients with PPI have a larger diameter of the aortic sinus (22), thinner IVS and LVPW, and larger diameter replacement valves (23), which are more common in PAR; The larger valve compresses the thinner membranous septal atrioventricular bundle, thereby inducing mechanical damage and increasing the risk of PPI after TAVI (24). On the contrary, in patients without PPI, thicker IVS and LVPW, and smaller diameter replacement valves, which is more common in PAR. The smaller valve compresses the thicker membranous septal atrioventricular tract, resulting in less mechanical damage and reducing the risk of pacemaker implantation after TAVI (25).

Different types of implanted aortic valves can have varying effects on cardiac conduction blocks. For the balloon expandable valves (BEV) and self-expanding valves (SEV), the rate of new LBBB post-TAVR is reported to be approximately 27% for the SEV CoreValve system and 11% for the BEV Edwards valve (26). In terms of PPI, the incidence of PPI is lower for BEV compared to SEV (OR 0.50, 95% CI 0.32 to 0.79) in patients with AS (27). However, for patients with PAR undergoing TAVI, the rates of new PPI can be varied. It is reported to be 16.35% for self-expanding CoreValve (28), 35.1% for balloon expandable Sapien3 (4), 2.3% for J-Valve (29), and 20% for Jena valve prosthesis (30). Interestingly, previous studies indicate a lack of reduction, or even an increase, in the rate of conduction system disturbances associated with these new-generation valves (26, 31, 32). This could potentially be explained by the continuous radial force exerted by the nitinol stent in SEV and the possibility of deeper implantation, leading to mechanical compression and injury to the His bundle (33, 34). In the case of VitaFlow Liberty^™^, a repositionable BEV, the incidence of PPI is higher in patients with PAR compared to those with PAS. This difference may be attributed to the incorporation of an external fabric cuff in the inferior part of the valve, which is intended to minimize paravalvular leak but may result in greater mechanical damage to the His bundle in patients with PAR.

## Conclusion

LBBB and AVB are the most common cardiac conduction blocks of TAVI with VitaFlow Liberty system, PAR is an independent risk factor for PPI in patients with aortic valve diseases undergoing TAVI, and PR > 200 ms is the most sensitive indicator for PPI after TAVI procedure.

## Limitations

Although the study offers valuable insights, it is important to acknowledge certain limitations. Firstly, the small sample size may restrict the generalizability of the results. Secondly, the single-center design could introduce bias. Conducting multi-center studies could yield more comprehensive data and validate our findings. Thirdly, as a retrospective study, there may be potential for selection and information bias, and establishing cause-and-effect relationships can be more challenging. Lastly, we lack follow-up data on conduction recovery one month or longer after TAVI with PPI. These limitations underscore the necessity for larger, multi-center, prospective studies with rigorous data collection to confirm our findings.

## Data Availability

The raw data supporting the conclusions of this article will be made available by the authors, without undue reservation.
